# The persian version of the fear-avoidance beliefs questionnaire among iranian post-surgery patients: a translation and psychometrics

**DOI:** 10.1186/s40359-024-01884-6

**Published:** 2024-07-15

**Authors:** Hamid Sharif-Nia, Erika Sivarajan Froelicher, Amir Hossein Shafighi, Jason W. Osborne, Reza Fatehi, Poorya Nowrozi, Bita Mohammadi

**Affiliations:** 1 Psychosomatic Research Center, Mazandaran University of Medical Sciences, Sari, Iran; 2 Department of Nursing, Amol Faculty of Nursing and Midwifery, Mazandaran University of Medical Sciences, Sari, Iran; 3 Department of Physiological Nursing, School of Nursing, Department of Epidemiology & Biostatistics, School of Medicine, University of California San Francisco, San Francisco, CA USA; 4 Department of Statistics, Miami University, Oxford, OH USA; 5 Student Research Committee, Mazandaran University of Medical Sciences, Sari, Iran; 6 Master of Nursing, Hospital Nurse 17 Shahrivar Amol, Mazandaran University of Medical Sciences, Sari, Iran

**Keywords:** Fear-avoidance, Avoidance beliefs, Validity, Reliability, Psychometrics, Persian

## Abstract

**Introduction:**

Fear-avoidance beliefs (FAB) play a crucial role in the treatment outcomes of post-surgery patients. These beliefs can lead to activity avoidance, increased pain, and decreased quality of life. Therefore, accurately measuring these beliefs in Iranian patients is of significant importance. The Fear-Avoidance Belief Questionnaire (FABQ) is a patient-reported questionnaire that evaluates individuals’ FAB. Since the validity and reliability of the Persian version of FABQ (FABQ-P) have not been assessed based on the Iranian population and sociocultural contexts, the current study has been implemented to determine the reliability and validity of the FABQ-P among Iranian post-operative patients by translation and psychometric properties.

**Methods:**

This methodological study conducted in 2023, a sample of 400 patients who had undergone surgery were selected using a convenience sampling method. The scale used in the study was translated and its psychometric properties were evaluated through network analysis and assessments of construct validity (including exploratory and confirmatory factor analysis), convergent validity, and discriminant validity. Additionally, the study assessed the internal consistency of the scale.

**Results:**

The MLEFA results with Promax and Kaiser Normalization rotation yielded two factors explaining 57.91% of the variance, encompassing 13 items. Also, the model was approved by CFA. Convergent and discriminant validity have been confirmed through the following criteria: Average Variance Extracted (AVE) exceeding 0.5, Composite Reliability (CR) surpassing 0.7, and Heterotrait-Monotrait Ratio of Correlations (HTMT) equating to 0.597. As for reliability, Cronbach’s alpha, composite reliability (CR), and MaxR for all constructs were greater than 0.7, demonstrating good internal consistency.

**Conclusion:**

As demonstrated by the results, the FABQ-P has a satisfactory level of reliability along with authentic validity according to the sociocultural contexts of Iranian post-operative patients.

**Supplementary Information:**

The online version contains supplementary material available at 10.1186/s40359-024-01884-6.

## Introduction

The Fear-Avoidance Belief (FAB) is a cognitive, emotional, and pain-oriented process in which an individual develops a strong belief that certain activities or circumstances are painful, detrimental, or threatening, leading to a fear response and subsequent avoidance behaviors toward those activities or circumstances [1, 2]. The process of FAB typically involves a recurring pattern or cycle of inauspicious thoughts, emotions, and behaviors [3, 4]. To illustrate, an individual may experience initial pain or discomfort while engaging in a specific movement or activity, which leads to apprehension and concern about any further potential harm [5, 6]. Following the avoidance behaviors, “physical discomfort” (in the form of increased muscle tension, fatigue, and pain) due to the sedentary activity [7], “psychological distress” (such as phobia, anxiety, and depression) caused by negative harm-related thoughts [8] and “chronic pain syndrome” [9] are expected. These FAB-related consequences may have outstanding implications for medical cooperation and adherence among patients, in the form of disobedience attitudes and impaired compliance toward medical care and treatment [10, 11]. A warning circumstance that its significance and concern are amplified when avoidance behaviors are associated with post-operative considerations [12], as the non-compliance attitudes toward post-operative considerations are significantly associated with a deceleration in the recovery process [10, 13] along with the life-threatening ramifications such as a Deep Venous Thrombosis (DVT) or Pulmonary Embolism (PE) [14, 15]. Adherence to post-operative guidelines, such as considering early physical activities is recommended as one of the most fundamental preventive recommendations for DVT and PE, which can be overlooked by patients because of their FAB [16–18].

The FAB is a complex and multifaceted concept, with a variety of factors contributing to its development, including personal experiences, social learning, cultural dissimilarities, and psycho-cognitive functions, among which, the sociocultural factors are noteworthy [3, 6, 19]. Sociocultural factors are derived from two comprehensive concepts of “culture” and “society”. Culture encompasses a diverse array of components that are contributed to conceptualizing the identity and shared experiences of a specific community, including the “values”, “norms”, “symbols”, “language”, “beliefs”, “ethics” and “religions” [20, 21]. Furthermore, social factors that contribute to the concept of “society” encompass various aspects of an individual’s social environment that have the potential to impact their attitudes, beliefs, behaviors, and health outcomes. These factors include cultural norms, social networks, family dynamics, socioeconomic status, and access to resources and support systems [22, 23]. Therefore, sociocultural factors can determine how FAB is perceived, experienced, regulated, and demonstrated by individuals. As mentioned earlier, FAB is closely correlated with the concept of “pain” [6, 24], which like FAB, is influenced by sociocultural factors [25]. Therefore, according to the various sociocultural contexts, enduring pain may be recognized as a value in some contexts, while others may prioritize demanding comfort [26–28]. Sociocultural context refers to the social and cultural factors that influence how we think and behave. These factors are shaped by interactions with society and culture, and are influenced by social institutions. In cognitive theory, sociocultural context plays a key role in shaping cognitive development and learning. Understanding and considering these contexts is important in fields like education, disability studies, and cognitive psychology [29–32].

Since Iran comprises various cultural, racial, ethnic, and religious populations, FAB among Iranians cannot be limited to a homogenous and monotonous setting and concept. Nevertheless, the majority of Iranians are Persian and Muslim [33, 34]. Iranians’ firm belief in fate and predestination in Islam, along with their obedience to Divine commandments, may have led them to believe that whatever happens in their lives is predetermined and based on God’s will [35, 36]. Being surrendered to God’s will can modulate the interpretation of Muslim Iranians towards pain and potential post-operative harm, as these concepts are often interpreted as a spiritual test of faith and patience, meaning that enduring pain and harm with patience and seeking relief through worship may result in spiritual and divine remunerations [37–39]. Moreover, based on the Iranian social norms, there is a stigma associated with being perceived as weak or dependent on others’ assistance. Hence, FAB among Iranian individuals may be due to their concerns about being judged by others, along with their inclinations toward independence and self-reliance [40–42]. Therefore, in congruence with what has been mentioned, sociocultural factors play a meaningful role in the exclusive conceptualization of FAB among Iranian post-operative patients.

Two main instruments assess the FAB; the “Avoidance Endurance Questionnaire (AEQ)” evaluates an individual’s ability to cope with what they avoid [43], and the “Fear-Avoidance Beliefs Questionnaire (FABQ)” which measures the level of FAB in individuals with chronic pain [44]. In addition to these mentioned instruments, the Tampa Scale for Kinesiophobia (TSK), Fear of Pain Questionnaire (FOPQ), Pain Catastrophizing Scale (PCS), and Pain Anxiety Symptoms Scale (PASS) can also be considered based on the pain-oriented aspects of FAB, the instruments which all generally concentrate on avoidance behaviors related to fear and pain [45–48]. FABQ is specifically designed to measure fear-avoidance beliefs related to physical activities and work. This specific focus on fear-avoidance beliefs in relation to particular activities distinguishes it from other questionnaires [49]. Other questionnaires such as TSK and FOPQ also address fear and avoidance, but FABQ particularly targets the relationship of these beliefs with work and physical activities [50]. Also, PCS focuses on catastrophic thinking related to pain and emphasizes negative and exaggerated beliefs about pain [51].

FABQ is a self-report questionnaire designed by Waddel et al. in 1993 in order to evaluate an individual’s beliefs about how physical activity and movement may potentially be accompanied by pain. FABQ comprises two subscales, including “work-related FAB” (measures an individual’s beliefs about how their work may affect their pain), and “physical activity-related FAB” (measures an individual’s beliefs about how physical activity might affect their pain). These factors had high internal consistency scores of 0.88 and 0.77, and together they accounted for 60.2% of the total variance in responses [44]. The utilization of the FABQ has shown remarkable effectiveness in clinical settings, particularly in assessing patients with acute or chronic physical pain, including those in post-operative situations. Moreover, its convenient yet comprehensive features, along with its concentration on psychological aspects of avoidance behaviors, set it apart from other similar instruments, making it the superior choice for evaluating FAB among patients with various pain conditions [44, 52–54]. The psychometric properties of the FABQ have been assessed in various studies. Accordingly, in the original study by Waddell et al. (1993), the FABQ demonstrated good internal consistency with Cronbach’s alpha values of 0.88 for the work subscale and 0.76 for the physical activity subscale. This indicates that the items within each subscale are highly correlated and measure the same underlying construct [40]. Moreover, the FABQ has been found to have good test-retest reliability, with intraclass correlation coefficient (ICC) ranging from 0.80 to 0.95 for both subscales [55]. Furthermore, it has shown good construct validity, as evidenced by its correlations with measures of pain intensity, disability, and other psychological constructs [40]. Eventually, studies have found that the FABQ is able to discriminate between individuals with high and low levels of fear-avoidance beliefs, supporting its validity as a measure of this construct [56]. Eventually, it is necessary to indicate that FABQ has been translated and psychometrically evaluated in various languages, populations, and cultures, including Italians [57], Japanese [58], Chinese [59], Arabic [60], and Finnish [61].

As previously declared, specific sociocultural factors can determine the development and manifestation of FAB among various populations and contexts, including Iranians. Therefore, considering the translation and psychometrics of FABQ in accordance with Iranian sociocultural contexts and metrics can be worthwhile in identifying FAB-related factors among Iranian post-operative patients, along with facilitating the implementation of its risk-reduction strategies. Besides, the validity and reliability of the Persian version of FABQ (FABQ-P) have not yet been assessed among Iranian populations. It is important to validate a questionnaire in the specific population of Iran to ensure its reliability and validity. Cultural differences can impact how individuals interpret and respond to questionnaire items, so the Persian language version of the instrument needs to be validated. This re-examination is necessary to ensure the questionnaire accurately measures the intended constructs in Iranian samples and supports its use in research and clinical contexts in Iran [62]. By conducting this validation, we contribute to the ongoing validation of the questionnaire and support its cross-cultural application. Therefore, the objective of the present study was to determine the reliability and validity of FABQ-P among Iranian post-operative patients through translation and psychometric properties.

## Methods

This methodological cross-sectional study was carried out between October to December 2023. Patients from Mazandaran (17 Shahrivar Hospital) (Amol, Iran) were recruited for this study.

### Inclusion and exclusion criteria

The inclusion criteria for participants in the study were: being at least 18 years old, being able to communicate in Farsi and being literate, volunteering to participate, and being hospitalized in hospital wards after surgery. Exclusion criteria included (based on their medical history and self-reported information during the initial screening process): 1- Cognitive Disorders, Reduced Level of Consciousness: These conditions could impair the patient’s ability to understand and accurately respond to the questionnaire items, compromising the validity of the data, 2- Presence of Mental Illness (e.g. Schizophrenia, Anxiety Disorder): Mental health conditions could independently influence the patient’s fear-avoidance beliefs and responses, confounding the assessment of the questionnaire’s psychometric properties in the post-surgery population, 3- Heart Diseases (e.g. Unstable Angina), Cerebrovascular Diseases, Neurological Diseases, Rheumatoid Arthritis: These medical conditions could directly impact the patient’s physical function and pain experience, factors that the Fear-Avoidance Beliefs Questionnaire aims to assess. Including such patients could introduce additional sources of variability, 4- Pregnancy, Cancer/Malignancies: These conditions would likely require specialized medical management that could affect the patient’s physical and psychological status, making them unsuitable for inclusion in a validation study focused on post-surgery patients, and 5- Drug Addiction, Drug Dependencies: Substance abuse issues could independently influence the patient’s pain perception, coping mechanisms, and questionnaire responses, confounding the assessment of the FABQ’s performance.

MacCallum et al. (1999) recommended a sample size of at least 200 cases for psychometric studies [63]. So, we decided to extend an invitation to 400 people due to the necessity of two different samples for construct validity. These 400 people gathered with the convenience sampling method. Following a thorough explanation of the study’s objectives, the participants were given questionnaires to fill out. The researcher collected questionnaires in person, explained the study, and invited participants to voluntarily take part. The researcher stayed with participants while they completed the questionnaires to ensure a high response rate and address any questions or issues. This approach helped prevent missing data and ensured immediate collection of completed questionnires.

### The original version of the questionnaire

Waddell et al. developed the Fear-Avoidance Belief Questionnaire (FABQ) to measure such beliefs in patients with low back pain. It is a 16-item, self-report questionnaire, in which each item is graded on a 7-point Likert scale (‘strongly disagree’ to ‘strongly agree’) and has two subscales shown by factor analysis, one subscale focusing on patients’ beliefs about how physical activities affect their pain and the other focusing on patients’ beliefs about how work affects their pain. The total score for the FABQ/pa ranges from 0 to 24, and the total score for the FABQ/w subscale ranges between 0 and 42. Internal consistency using Cronbach’s alpha for two subscales was obtained as 0.88 and 0.77 respectively [44]. Two phases were used to assess the psychometric qualities and usefulness of the “Persian version of the Fear-Avoidance Belief Questionnaire (FABQ -P)”.

### Translation

To conduct this study, we secured written permission from the Questionnaire’s developer to use the FABQ. Subsequently, the Questionnaire was translated from English to Persian following the Gudmundsson [64] translation protocol. Two proficient English-Persian translators independently translated the FABQ into Persian. An expert panel, comprised of some of the authors of this article and two professional translators, meticulously reviewed and amalgamated the two translations to create a Persian version of the FABQ. Subsequently, a Persian-English translator was engaged to translate the FABQ-P back into English. The panel of experts reviewed and approved this final version.

### B. Phase II

#### Face validity

In order to determine the face validity of the FABQ, it was given to 10 patients who fit the criteria. Experts then shared their opinions on the content, clarity, readability, simplicity, comprehensibility of the questions, and how easy it was to complete the questionnaire.

#### Content validity

Experts in the fields of psychiatry and psychology were asked to review and provide feedback on the FABQ. They focused on evaluating word choice, grammar, the relevance and importance of items, their order, and scoring accuracy. Minor grammatical errors were fixed based on their suggestions.

### Normal distribution, outliers, and missing data

Skewness (± 3) and kurtosis (± 7) were used to individually investigate the univariate distribution of data. Also, multivariate normality distribution was assessed by the Mardia coefficient of multivariate kurtosis (< 8). Mahalanobis d-squared (*p* < 0.001) was used to determine whether there were any multivariate outliers [65]. The missing data were assessed using multiple imputations, and and exploratory factor analysis used the pairwise deletion method to handle missing data.

### Construct validity

To test the construct validity, the original dataset (*n* = 400) was randomly divided into two datasets with 200 cases each. With the first random dataset (*n* = 200), the Maximum Likelihood Exploratory Factor Analysis (MLEFA) with Promax with Kaiser normalization rotation was conducted to determine the factor structure. The Kaiser–Meyer–Olkin (KMO) > 0.8 and Bartlett’s test of sphericity to be significant (*p* < 0.01) were referred to ensure the data was relevant and appropriate for performing the factor analysis. The factorability of the data was evaluated using a combination of criteria, including eigenvalues exceeding 1, communalities greater than 0.2, and factor loadings exceeding 0.3, in conjunction with a visual inspection of the scree plot to determine the optimal number of factors to retain [66–68]. Also, the exploratory factor analysis was performed using parallel analysis to determine the optimal number of factors to extract, providing a data-driven approach to identifying the underlying structure of the variables [69]. The eigenvalues (λ) were computed as the sum of squared factor loadings (SSL) across all items (k) for each factor, representing the proportion of variance in each item that can be attributed to the factor. The eigenvalue was then divided by the total number of items to determine the percentage of total variance explained by each factor [70]. The MLEFA was performed using SPSS version 27.

In the next step, the factor structure obtained from MLEFA was analyzed and confirmed by conducting Confirmatory Factor Analysis (CFA) based on the second random dataset (*n* = 200) using AMOS version 27. The following model fit indices were used to assess the model fit: Comparative of Fit Index (CFI) was > 0.9, Normed Fit Index (NFI) was > 0.9, Tucker-Lewis Index (TLI) was > 0.9, and Incremental Fit Index (IFI) was > 0.9; that of Root Mean Square Error of Approximation (RMSEA) was < 0.08; and for Minimum Discrepancy Function divided by degrees of freedom (CMIN/DF) < 3 was considered good [71].

### Convergent validity and discriminant validity

In order to evaluate the convergent validity, we followed the methodology proposed by Fornell and Larcker (1981). This involved calculating the average variance extracted (AVE) and composite reliability (CR) for each construct. Convergent validity is considered to be present when the AVE for a construct is greater than 0.50, indicating that the construct accounts for more than half of the variance in its indicators on average. Also (CR) should be greater than 0.7 [72]. For the assessment of discriminant validity, we employed the heterotrait-monotrait (HTMT) ratio of correlations, as suggested by Henseler, Ringle, and Sarstedt (2015). The HTMT ratio compares the average of the heterotrait-heteromethod correlations to the average of the monotrait-heteromethod correlations. A value below 0.90 signifies that divergent validity has been established between two reflective constructs [73].

### Reliability

The Cronbach’s alpha, McDonald’s omega (Ω), average inter-item correlation coefficient (AIC), Composite Reliability (CR), and Maximal Reliability (MaxR) were calculated to gauge the internal consistency and construct reliability. If the α, Ω, CR, and MaxR were greater than 0.7 and AIC values of 0.2 to 0.4 were interpreted as acceptable internal consistency [74, 75].

### Feasibility and acceptability

In order to assess feasibility and acceptability, we analyzed the time it took to complete the questionnaire along with various psychometric indicators. On average, participants spent 10 to 15 min completing the questionnaires.

### Fear-avoidance belief score

Descriptive statistics were employed to calculate the mean score of Fear-Avoidance Belief. Additionally, an independent samples t-test was conducted to evaluate differences between the groups of men and women with respect to Fear-Avoidance Belief.

## Results

### Demographic characters

The mean age of the participants was 44.38 (SD = 13.49) years. Among the 400 participants, 152 (46.1%) were women and 178 (53.9%) were men, 99 (24.75%) were single and 301 (75.25%) were married, and 120 (30%) participants had a history of surgery, and the type of admission of 300 (75%) participants was emergency.

### The results of MLEFA

The results of MLEFA with Promax with Kaiser Normalization rotation using the first random dataset (*n* = 200) extracted two factors accounting for 57.91% of the variance comprising 13 items. Item 4, item 12, and item 13 were removed from the original version due to communalities of less than 0.2, and factors loading of less than 0.5. Moreover, the results of the KMO (0.899) and Bartlett’s test of sphericity (*p* < 0.001, Chi-square = 3336.497, *df* = 78) showed the sampling is adequate and appropriate for conducting the factor analysis. The detailed results of the MLEFA are shown in Table 1 (The result of MLEFA on the two factors Persian version of the Fear-Avoidance Beliefs (*n* = 200)).

**Table 1:** The result of MLEFA on the two factors persian version of the fear-avoidance beliefs (*n* = 200)

Factor	Items	Factor loading	h^2^	λ	% Variance
Work-Related Pain and Activity Limitations	**Q**_**7**_. My work aggravated my pain	0.957	0.821	5.987	46.05%
	**Q**_**10**_. My work makes or would make my pain worse	0.935	0.822		
	**Q**_**9**_. My work is too heavy for me	0.842	0.640		
	**Q**_**5**_. I cannot do physical activities which (might) make my pain worse	0.823	0.677		
	**Q**_**2**_. Physical activity makes my pain worse	0.806	0.662		
	**Q**_**14**_. I cannot do my normal work till my pain is treated	0.757	0.584		
	**Q**_**8**_. I have a claim for compensation for my pain	0.690	0.473		
	**Q**_**11**_. My work might harm my back	0.613	0.637		
	**Q**_**3**_. Physical activity might harm my back	0.498	0.471		
	**Q**_**6**_. My pain was caused by my work or by an accident at work	0.496	0.475		
	**Q**_**1**_. My pain was caused by physical activity	0.493	0.245		
Work Ability Prognosis	**Q**_**15**_. I do not think that I will be back to my normal work within 3 months	0.889	0.779	1.542	11.86%
	**Q**_**16**_. I do not think that I will ever be able to go back to that work	0.867	0.686		

*Abbreviations* h^2^: Communalities, λ: Eigenvalues

### The results of CFA

**Fig. 1:**
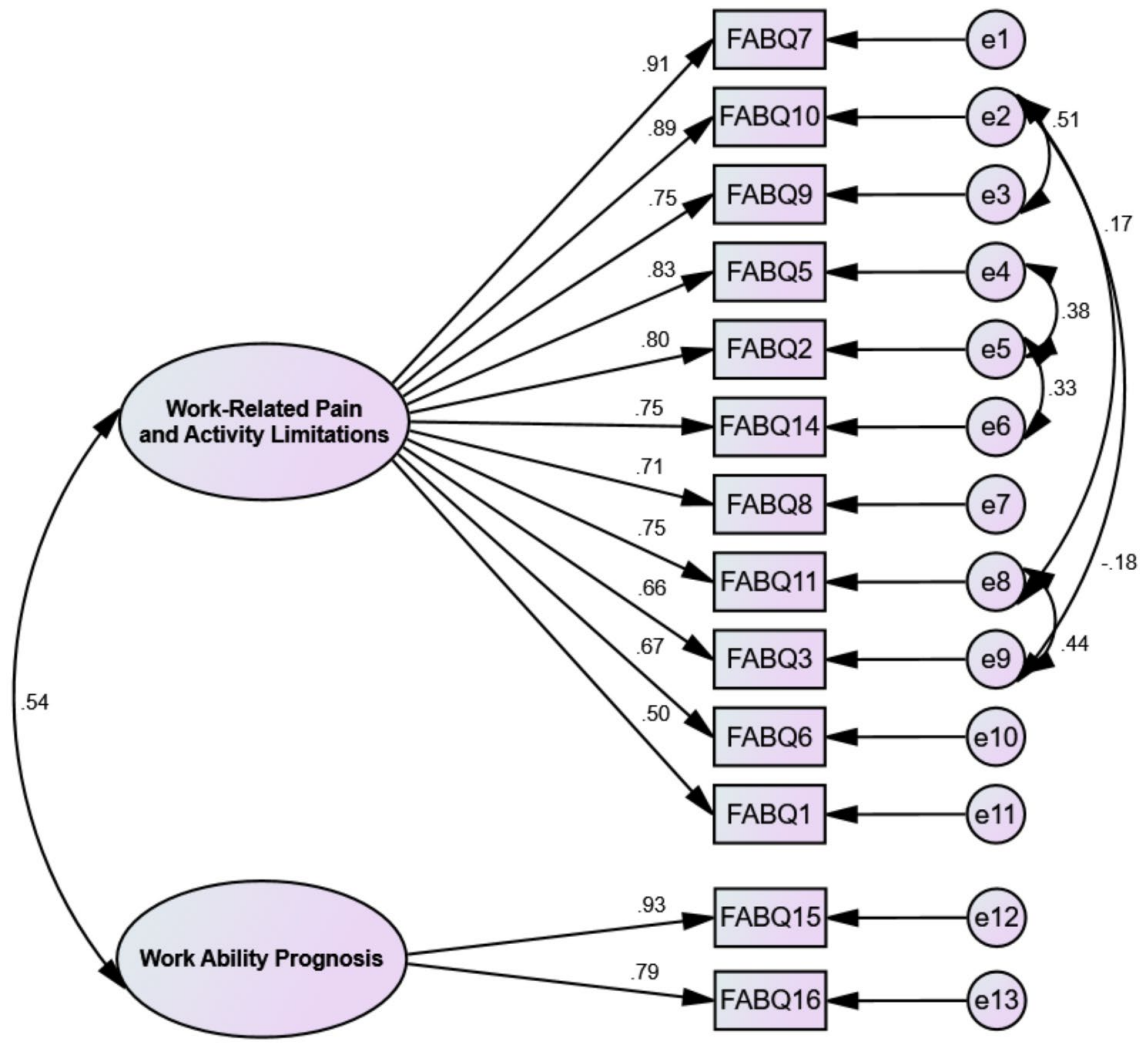
The results of the CFA and factor loadings

The CFA was conducted to confirm and validate the factor structure obtained from MLEFA (*n* = 200) using the second random dataset (Fig. 1). The initial results showed that the data fited the model well as evidenced by (*χ*^2^ [58] = 155.220, *p* < 0.001, *χ*^2^/*df* = 2.676, CFI = 0.927, NFI = 0.912, IFI = 0.928, TLI = 0.902, RMSEA = 0.064.

### Convergent and discriminant validity

Table 2 (The results of the convergent validity and construct reliability (*n* = 200)) shows the results of the CFA. The results showed that AVE for factors of Work-Related Pain and Activity Limitations and Work Ability Prognosis was greater than 0.5, indicating good convergent validity. Hence, with the factors of Work-Related Pain and Activity Limitations and Work Ability Prognosis, CR greater than 0.7, it can be concluded that convergent validity for all constructs has been established. As for discriminant validity, the results of the HTMT ratio showed that the correlation between Work-Related Pain and Activity Limitations and Work Ability Prognosis (0.597), was lower than 0.85, demonstrating good discriminant validity for all constructs.

**Table 2:** The results of the convergent validity and construct reliability (*n* = 200)

Factors	α	Ω	CR	MaxR	AVE	AIC
Work-Related Pain and Activity Limitations	0.918	0.919	0.921	0.936	0.521	0.566
Work Ability Prognosis	0.836	0.836	0.856	0.886	0.749	0.741

*Abbreviations* α: Cronbach’s alpha, Ω: McDonald’s omega

### Reliability

As for reliability, the Cronbach’s alpha, McDonald’s omega, CR, and MaxR for all constructs were greater than 0.7, and AIC values of 0.2 to 0.4 were interpreted as acceptable internal consistency.

### Fear-avoidance belief score

In the overall population, the mean score for fear-avoidance Belief was 47.26 (SD = 24.98, 95%CI: 44.56, 49,97). Furthermore, there were no significant differences (*p* = 0.569) in Fear-Avoidance Belief scores between men (46.53, SD = 24.13) and women (48.12, SD = 26.00).

## Discussion

The main aim of the current study was to conduct a translation of the FABQ into Persian and assess its reliability and validity through psychometric analysis among the samples of Iranian post-operative patients. Following a comprehensive evaluation, the results demonstrated satisfactory values for factor structure, validity, and reliability of the FABQ-P.

The current study, which assessed the structural validity of the FABQ-P, has demonstrated that 46.05% and 11.86% of the total variance were accounted for by the first (11 items) and second (2 items) factors, respectively. Moreover, the combination of these mentioned two factors yielded a total variance of 57.91%. Accordingly, these statistical findings confirm the validity of the FABQ-P as an appropriate instrument for assessing FAB among Iranian post-operative patients. Furthermore, similar previous studies have illustrated that the FABQ, which was originally developed by Waddell et al. in 1993 [44], has been successfully translated and psychometrically evaluated in multiple languages and various sociocultural contexts. Subsequently, all mentioned studies have demonstrated consistent validity, despite variations in methodology [57–61]. This demonstrates the robustness and generalizability of the FABQ across diverse populations. In comparison to other articles in the literature, the current study adds to the growing body of research supporting the validity of the FABQ in assessing FAB in post-surgery patients. The findings contribute to our understanding of the psychometric properties of the FABQ-P and its applicability in a specific cultural context, while also emphasizing the importance of cross-cultural validation studies to ensure the reliability and validity of assessment tools across different populations.

The concept of FAB within Iranian post-operative patients has been defined as the two fundamental factors or terms of “Work-Related Pain and Activity Limitations (WRPAL)” and “Work Ability Prognosis (WAP)”. Factors that concentrated on the causal relationship of “pain, fear, avoidance, and concern due to the activity and work”, which, beyond their body-oriented attributes, their correlated psycho-cognitive process have been deemed more conspicuous.

The initial factor was the concept of WRPAL, which refers to physical discomfort or pain experienced by an individual due to their work, activities, tasks, or duties [76, 77]. The WRPAL can hinder individuals’ capability to perform certain expected activities, along with leading them to restricted productivity, efficiency, and expediency [78, 79]. In this particular context, individuals with FAB associated with WRPAL may decide to restrict their physical activity to avoid experiencing potential pain and limitations arising from their work and activities. In essence, individuals hold the belief that involvement in specific tasks or activities may intensify their pain or result in further harm. Hence, they opt to abstain from them, despite their essential and indispensable nature. A hypothesis that has accrued substantial support through many investigations [80–83]. Nevertheless, within the Iranian sociocultural context and setting, there is a prevailing emphasis on diligence and efficacy, alongside the enduring of suffering or discomfort [84, 85]. However, apprehensions about exacerbating pain may compel individuals to refrain from engaging in specific endeavors, thereby culminating in fear-avoidance behavior [6, 86]. In light of the prominence of work-related FAB subscales in FABQ, it appears that “work” was a predominant mental preoccupation among participants in this study. Analysis of the “work” subscale of FABQ suggests that many participants perceived their work as challenging, leading to significant pain and discomfort. Consequently, these individuals developed a cognitive association between physical pain and the demands of their work, which in turn influenced their post-operative FAB. These results are consistent with previous research that has demonstrated a strong link between work-related factors and FAB in individuals with musculoskeletal conditions [56].

The subsequent factor, which has been elucidated as the concept of WAP, involves forecasting an individual’s capacity to carry out work duties in the future based on various factors including health, functional capacity, skills, and occupational demands. WAP, which is often discussed in the context of occupational health and rehabilitation, encompasses evaluating the likelihood that an individual will be able to continue working at their current or even in a different occupational status, as well as identifying potential barriers or challenges that may impact their capability to work [87, 88]. As previously declared, individuals who hold a FAB may be less likely to actively engage in post-operative instructions [10–18]. Such reluctance to cooperate is typically driven by apprehensions surrounding experiencing pain or exacerbating injury, ultimately elongating the recovery period and increasing the risk of complications, which could eventually result in occupational loss or inability to maintain previous occupational performance [89, 90]. Furthermore, the mentioned concern could be aggravated due to the unstable economic circumstances of Iran, as the country has faced enormous economic challenges in recent years, including high inflation and unemployment rates [91, 92]. Overall, what has been proposed in relation to WAP aligned with previously indicated hypotheses concerning the concept of WRPAL. The sequence of correlations among the concepts of “challenging work”, “interpreting pain through previous work-related experienced pain”, “development of FAB,” and ultimately “apprehension regarding the potential loss of such challenging work” has been established.

The outcomes of confirmatory factor analysis demonstrate that the proposed model in this study aligns closely with the obtained data, indicating strong support for the two-factor conceptual framework. Specifically, both factors exhibited a significant correlation with the overall FAB score, highlighting the coherence and validity of the established model. Regardless of some methodological differences, these findings are consistent with previous investigations that have also supported the two-factor structure of the FABQ, [93, 94].

Moreover, within the present investigation, Cronbach’s alpha values of 0.91 and 0.83 were established for the factors of WRPAL and FAB, respectively. This signifies that the components derived by the FABQ-P are assessing a cohesive concept, exhibiting favorable levels of precision, dependability, and repeatability. Thus, the consistent reliability demonstrated by translations of various editions of the FABQ underscores its robustness in diverse linguistic settings [57–61]. The consistent and robust reliability of the FABQ across different translations underscores its utility and effectiveness in assessing FAB among patients with physical injuries. This further strengthens the validity and applicability of the FABQ-P in clinical practice and research settings. In fact, comparisons with other similar questionnaires in the literature further support the superior psychometric properties of the FABQ-P, highlighting its value as a reliable measurement tool in the assessment of post-surgery related FAB.

The findings of the present investigation suggest that Iranian patients who have undergone operation exhibit a moderate level of FAB, with no notable disparity between the genders. In other words, in the Iranian context, it is believed that gender diversity did not have a significant impact on the experience of FAB among both male and female patients. The outcomes of the current study exhibited notable variances from prior studies conducted in disparate linguistic settings, in the form of the manifestation of FAB and demographic disparities which may be attributed to discrepancies in research methodologies as well as varying cultural influences and contextual indicators [57–61]. The aforementioned assertions were also aligned with the framework of Iranian sociocultural metrics, as elucidated extensively in relation to the theoretical concepts of WRPAL and FAB [37–42]. For example, most Western cultural contexts revealed higher levels of FAB among male patients compared to female patients. This disparity was attributed to differences in societal expectations and gender roles [95–97].

In summary, the results of this study underscored the remarkable influence of intricate sociocultural elements on the development of FAB among Iranian post-operative patients, shedding light on the complexities involved in defining and navigating the concept of FAB within the mentioned population. This is consistent with previous investigations that have demonstrated the role of sociocultural factors in shaping patients’ attitudes towards pain and recovery [98, 99]. Recognizing the paramount importance of this assertion is crucial as it underscores the potential for a more nuanced understanding of FAB to empower healthcare providers in effectively addressing patients’ concerns, fostering greater patient engagement in post-operative care, and ultimately mitigating the potential adverse consequences of post-operative circumstances.

### Limitations and strengths

The study was conducted only among post-operative Iranian patients, so the results may not apply to other clinical circumstances. Additionally, since the population of the current study was limited to Amol (a city in the Mazandaran province of Iran), generalizing its results to the entire Iranian population may be affected, as Iran is a multi-sociocultural country. Moreover, the data collection process during the current study was limited to a single point in time, so it is not possible to determine how FAB may change over time. Reciprocally, despite its novelty, the implementation of exploratory graph analysis to recognize factors associated with FAB, along with the calculation of the Omega-McDonald’s coefficient and Cronbach’s alpha, has been considered as other significant strengths.

### Implications

The translation and psychometric testing of the Persian Version of the FABQ among Iranian post-operative patients could reveal the impact of sociocultural differences on the concept of FAB. By adapting the questionnaire to the Persian language and Iranian sociocultural context, healthcare providers in Iran can better understand and address FAB in post-operative patients, leading to improved diagnostic and therapeutic services. Furthermore, testing the translated questionnaire can also ensure its reliability and validity in the multi-sociocultural Iranian population, ultimately enhancing its usefulness among clinical settings and allowing for targeted interventions to alleviate FAB-related post-operative consequences. To conclude, the results of the current study can consolidate the growing body of literature on FAB and its correlated consequences on post-operative recovery outcomes.

## Conclusion

Given our current knowledge, the present study has been at the forefront of assessing the accuracy and consistency of the concept of FAB among Iranian post-operative patients, taking into account factors such as WRPAL and WAP. Our findings reveal that the Persian version of FABQ demonstrates a sound structure and reliability within the Iranian sociocultural context and metrics. As a result, the FABQ-P can be considered as a valuable tool for healthcare professionals in understanding and addressing fear-driven avoidance behaviors among Iranian patients, encouraging their engagement in collaborative post-operative interventions and potentially reducing complications associated with FAB.

### Electronic supplementary material

Below is the link to the electronic supplementary material.

Supplementary Material 1

## Data Availability

The data that support the findings of this study are available from the corresponding author upon reasonable request.

## Abbreviations

FAB: The Fear-Avoidance Belief
DVT: Deep Venous Thrombosis
PE: Pulmonary Embolism
AEQ: Avoidance Endurance Questionnaire
FABQ: Fear-Avoidance Beliefs Questionnaire
TSK: the Tampa Scale for Kinesiophobia
FOPQ: Fear of Pain Questionnaire
PCS: Pain Catastrophizing Scale
PASS: Pain Anxiety Symptoms Scale
FABQ-P: The Persian version of the Fear-Avoidance Belief Questionnaire
MLEFA: Maximum Likelihood Exploratory Factor Analysis
KMO: The Kaiser–Meyer–Olkin
λ: The eigenvalues
SSL: Sum of squared factor loadings
CFA: Confirmatory Factor Analysis
CFI: Comparative of Fit Index
NFI: Normed Fit Index
TLI: Tucker-Lewis Index
IFI: Incremental Fit Index
RMSEA: Root Mean Square Error of Approximation
CMIN/DF: Minimum Discrepancy Function divided by degrees of freedom
CR: Composite reliability
AVE: Average Variance Extracted
HTMT: heterotrait-monotrait ratio
α: Cronbach’s alpha
Ω: McDonald’s omega
AIC: average inter-item correlation coefficient
MaxR: Maximal Reliability
WRPAL: Work-Related Pain and Activity Limitations
WAP: Work Ability Prognosis
h^2^: Communalities
